# Supplementary material for: Fracture strength analysis of titanium insert-reinforced zirconia abutments according to the axial height of the titanium insert with an internal connection
Source: PLoS One. 2021 Apr 1;16(4):e0249208. doi: 10.1371/journal.pone.0249208 (PMC8016344; doi:10.1371/journal.pone.0249208)
Supplement: Attachment — Submitted filename: Reponse letter for plos-one.docx [file pone.0249208.s002.docx]

<http://www.google.co.kr/url?sa=i&rct=j&q=&esrc=s&source=images&cd=&cad=rja&docid=VI0FkyAy_B2MMM&tbnid=DIJN1x1lf4AisM:&ved=0CAUQjRw&url=http://blog.daum.net/loveubrad/3&ei=MxmpUq2kJs2fiAf4t4CoAg&bvm=bv.57799294,d.aGc&psig=AFQjCNGPIXzBVP6rLjHPpnbHaf0LBMjETg&ust=1386900141258164>Dept. of Advanced General Dentistry

Yonsei University College of Dentistry

250 Seongsanno, Seodaemun-gu

Seoul 120-752, Republic of Korea

jby1004@yuhs.ac

Tel: +82-2-2228-8980

Fax: +82-2-2227-8906

1. Title:

REVISION: PONE-D-21-03699
Fracture strength analysis of titanium insert reinforced zirconia abutments according to the axial height of titanium insert with internal connection
2. Corresponding author’s name, mailing address, phone number and fax number:

Prof. Bock -Young Jung

E-mail address: [jby1004@yuhs.ac](mailto:gyjung@gist.ac.kr)

Phone: (+82)2-2228-8980, Fax: (+82)2-2227-8906

3. Names of authors:

Seung-rye Song, Kyeong-Mee Park, Bock-Young Jung

This response letter accompanies our online resubmission of the manuscript titled “ Fracture strength analysis of titanium insert reinforced zirconia abutments according to the axial height of titanium insert with internal connection ” for the publication in “ PLOS ONE ”.

We really thank the reviewer’s valuable comments which made our manuscript more sound and reasonable scientifically. We are re-submitting after revisions as requested by the reviewers. We tried to address all the comments carefully in the revised manuscript. We hope that our revised manuscript meets the high standards of your journal**.**

We hope that this paper would be published in ‘PLOS ONE’. We thank you in advance for your time and attention.

Sincerely yours,

4th March, 2021

Bock Young, Jung

Response letter

Manuscript ID: PONE-D-21-03699

MS-Type: Original Article

Title: " Fracture strength analysis of titanium insert reinforced zirconia abutments according to the axial height of titanium insert with internal connection."

Correspondence Author: Bock- Young, Jung

Reviewer #1

1. There is no specification of implant type regarding sterile and clinical usage type of implants, while those implants on the figures attached have smooth surface and they should not be used for clinical purposes, the surface should be rough and specific related to the manufacturer! This in vitro study should be performed on the original, sterile packed dental implants prepared for clinical use.

**Response:** We respect and appreciate the comment of the reviewer.

We have modified ‘material and methods’ section and specified the information related to the implants tested.

**Text Change**: See “Material and methods”.

“The implants tested in this study were composed of SLA surface-treated pure titanium and were consistent with those used clinically.”

2. English should be carefully checked by the native speaker and the manuscript need serious proof editing before final consideration for publication

**Response:**

Thank you for the comment. We carefully tried to correct any errors in spelling, grammar, and terminology of this manuscript.

**Text Change:** The corrected words and phrases were highlighted.

Reviewer #2

I have reviewed the manuscript Fracture strength analysis of titanium insert reinforced zirconia abutment by axial height of titanium insert with internal connection” submitted to “PLOS ONE” for publication. In this study, authors have analyzed the fracture strength in vitro by varying the axial height of the titanium insert and the labial height of the zirconia abutment in an internal connection implant to identify the titanium insert axial height with optimal mechanical stability. The manuscript fits well within the scope of the journal; it needs some major improvements; there are a few suggestions that authors may consider improving it further:

1. The use of English language is reasonable, however, there are a number of minor punctuation and grammatical errors; that should be corrected and rephrased using academic English for a better flow of text for reader.

**Response:**

We appreciated this comments and this manuscript was reedited by one of native experts whose specialty was dentistry according the advice of the reviewer.

**Text Change:** See the marked-up manuscript.

2. Introduction; is very detailed and covering the background information and the rationale of the study effectively. Some of the statements have missing citations; such as for this statement. A number of studies have proven the relevance…..: author may cite the following references. Similarly, the text in the second paragraph of the introduction require citations.

A titanium insert, as a complementary component….. require citations as given above can be used.
**Response:**

We appreciate this important comment of the reviewer. We have specified the references we cited.

**Text Change:** See “Introduction” section.

3. Methods: why not used same number of samples after exclusion (n=?) for each group

Gr1 (n=8), Gr2 (n=12), Gr3 (n=9), Gr4 (n=11), Gr5 (n=11).?

**Response:**

We appreciated this valuable comment of the reviewer.

This study was originally prepared by dividing 60 specimens into 5 groups, 12 pieces for each group. However, we excluded nine specimens. Four specimens during the preparation for screwing and thermocycling procedures and 5 specimens during the preparation for loading were failed, which could affect the results of the experiment.

**Text Change:** We have modified the end section of discussion and added statement on the reason for the uneven distribution of specimens.

4. In discussion: please include discussion in terms of reduced vertical height; or reduced inter occlusal space (deep bite) case. Is there any relation?
**Response**

Thank you for the valuable comment. According to the advice of reviewer, we have introduced and added more description regarding the clinical implications that the reduced interocclusal distance has.

**Text Change:** See “Discussion” in page.

“Vertical crown height or interocclusal space is also an important factor to consider when designing titanium insert reinforced zirconia abutments. The inter occlusal space means the distance between the ridge crest and the occlusal plane comprising the depth of the soft tissue above the fixture, the height of the abutment, and the thickness of the implant prosthesis [17]. Based on the results of this study that the axial height of the titanium insert should be 3mm or more to ensure structural integrity, the limited interocclusal space can be a contra-indication to titanium insert-reinforced zirconia prosthesis unless the margin of zirconia prosthesis is located subgingivally.”

5. What are the limitations of the study?

**Response:**

Thank you for the valid comment of reviewer. We have modified “Discussion” adding several limitations of this study and presented the next research for the further investigation.

**Text Change:** See “ Discussion” in page.

“ The uneven and small numbers of specimens per group (Gr 1 (8), Gr 2 (12), Gr 3 (9), Gr 4 (11), and Gr 5 (11)) is considered one of the limitations of this study. To avoid bias in the data analysis, nine failed specimens in the preparation stages for the screwing, thermocycling, and loading test were excluded. In addition, non-anatomical, internal connection type abutments were used in this study. Further experimental studies using anatomically designed zirconia prostheses and large-size specimens are required to achieve more clinically meaningful results. An experimental study is needed to investigate the fatigue load and labial thickness of titanium insert-reinforced zirconia abutments to maintain the integrity of the abutment complex under dynamic functional loading using a greater number of specimens and a load applicator to mimic the conditions of occlusion between anterior teeth, followed by supporting clinical research.“

Thank you very much again.

*We really thank the reviewer’s comments which really solidify our manuscript more reasonably.*

Sincerely,

Bock Young Jung

(Corresponding author)
